# Biomarkers in preclinical and early Alzheimer’s disease in China: a scoping review

**DOI:** 10.1016/j.tjpad.2026.100599

**Published:** 2026-05-19

**Authors:** Guoping Peng, Yan Yang, Ying Wang, Sagar Anil Chandekar, Jintai Yu

**Affiliations:** a Department of Neurology, First Affiliated Hospital, Zhejiang University School of Medicine, Hangzhou, Zhejiang, PR China; b Novo Nordisk (China) Pharmaceuticals Co., Ltd., Beijing, PR China; c Novo Nordisk A/S, Søborg, Denmark; d Department of Neurology, Huashan Hospital, Shanghai Medical College, Fudan University, Shanghai, PR China

**Keywords:** Biomarkers, Alzheimer's disease, Chinese population, Early diagnosis, Scoping review

## Abstract

This scoping review synthesizes evidence on fluid and neuroimaging biomarkers for preclinical and early Alzheimer’s disease (AD)—including mild cognitive impairment (MCI) and mild AD dementia—in Chinese populations, where a comprehensive overview has been lacking despite AD's increasing biomarker-based definition. We systematically searched four English databases (PubMed, EMBASE, Cochrane, and Web of Science) and three Chinese databases (CNKI, Wanfang, and CQVIP) for studies (2013–2023) reporting diagnostic accuracy of these biomarkers in Chinese preclinical and early AD (eAD) cohorts for clinical use. Due to rapid advancements in biomarker research in China, a supplementary search was conducted in the four English databases for studies published between 2024 and April 30, 2025. Of the 366 included studies investigating fluid or neuroimaging biomarkers in AD, 48 specifically evaluated biomarker performance in biomarker‑confirmed AD populations. Plasma p-tau217 showed strong performance for diagnosing MCI due to AD, and plasma p-tau217, p-tau181/Aβ42, and p-tau217/Aβ42 effectively classified amyloid-β (Aβ) pathology. Multimodal combinations and MRI-based biomarkers also performed well, though evidence is limited. In China, biomarker diagnosis of MCI due to AD is advancing rapidly, while approaches for preclinical AD, machine learning, and multi-protein panels remain in early development.

## Highlights

• Biomarker research for early stages of AD surged in the past five years in China.
• 86 high-performance biomarkers were identified in biomarker-confirmed AD patients.
• Most biomarker studies address diagnosis, with a subset for preclinical and MCI-AD.
• Seven biomarkers were well-validated across multiple Chinese AD cohorts for Aβ.
• Many innovative biomarkers are under active development in Chinese AD cohorts.

## 1 Introduction

Alzheimer's disease (AD) is a progressive neurodegenerative disorder characterized by cognitive decline, memory loss, and functional impairment, representing the most common cause of dementia worldwide [1]. The global burden of AD is staggering, with an estimated 55 million people affected, a number projected to 139 million by 2050 due to aging populations [2]. In China, the prevalence of AD is rising rapidly, with over 10 million cases reported, posing significant challenges to healthcare systems and caregivers [3]. Early diagnosis and intervention are critical to slowing disease progression, preserving cognitive function, and reducing the socioeconomic burdens associated with AD [4]. However, achieving early and timely detection, diagnosis, and intervention in AD remains a formidable challenge, particularly in resource-limited settings.

Traditionally, AD has been diagnosed clinically—a syndromic diagnosis based on symptoms, neuropsychological assessments, and exclusion of alternative causes, representing a probabilistic determination in the absence of biomarker confirmation. The landscape of AD diagnostics has been fundamentally reshaped by the advent of biomarkers, shifting from this clinical syndromic approach (diagnosis of exclusion) toward a biological diagnosis (diagnosis of inclusion), supported by biomarkers reflecting the underlying etiology or pathobiology of the disease. According to the 2024 revised Criteria for Diagnosis and Staging of Alzheimer's Disease by the Alzheimer's Association (AA 2024) framework, AD is now understood as a continuum encompassing preclinical, mild cognitive impairment (MCI), and dementia stages, with biomarkers playing a pivotal role in identifying each phase [5]. Incorporating AD biomarkers in the inclusion criteria of clinical trials enables the enrollment of patients with AD at earlier stages of the disease, in which there may be no functional impairment or even no detectable clinical abnormality. It provides healthcare professionals with the opportunity to intervene in the disease process early on, already before the onset of overt dementia.

The modalities of AD-related biomarkers are diverse throughout the entire AD disease continuum. According to the AA 2024 diagnostic and staging criteria, core 1 biomarkers reflecting amyloid-β (Aβ) pathology such as Aβ positron emission tomography (PET) and cerebrospinal fluid (CSF) Aβ42, as well as core 2 biomarkers reflecting phosphorylated tau pathology, such as secreted tau (CSF or plasma phosphorylated tau [p-tau]181, p-tau217 and p-tau231), can be used to support an AD diagnosis [6]. However, CSF biomarkers are limited by their invasiveness and patient discomfort in clinical practice [7]. Neuroimaging techniques, particularly PET, remain costly and inaccessible in many settings [8]. Plasma AD biomarkers have over the last decade been a major research focus. Beyond core biomarkers, biomarkers reflecting tau pathology (**e**ndogenously cleaved Microtubule-Binding Region [eMTBR]-tau243, p-tau205), neurodegeneration (t-tau, neurofilament light [NfL]), and neuroinflammation (plasma glial fibrillary acidic protein—plasma GFAP) demonstrate promise for use in multiple clinical contexts of AD—ranging from supporting diagnosis and staging to differential diagnosis, risk stratification, and therapeutic monitoring. Nonetheless, their utility warrants further exploration and validation in diverse clinical settings and patient populations.

Over the past decade, numerous studies in China have generated a wealth of valuable data on AD biomarkers [[9], [10], [11], [12], [13], [14]]. A number of studies have been conducted in Chinese AD or aging cohorts, with some of them involving participants with biomarker-confirmed AD, requiring evidence of amyloid positivity as determined by PET or CSF. These studies provide important evidence and practical insights into the application of novel biomarkers in diagnostically confirmed AD populations. To advance the early diagnosis and intervention of AD in China, it is critical to delineate the existing clinical evidence and identify gaps in the application of emerging biomarkers, particularly for diagnosing the early stages of AD, which include preclinical, MCI, and mild dementia due to AD as defined by the AA 2024 criteria. Biomarker evidence that has already been validated against FDA-approved and other regulatory-approved reference standards for accurately identifying patients with biomarker-confirmed AD is critical.

However, existing studies often focus on specific biomarkers or clinical scenarios, leaving gaps in understanding the broader landscape of biomarker applications and their clinical utility.

This scoping review aims to systematically map and characterize studies conducted in Chinese populations over the past decade that investigated fluid and neuroimaging biomarkers for clinical applications in preclinical AD and early AD (eAD). For the purposes of this review, eAD is defined as encompassing both MCI due to AD (MCI-AD) and mild AD dementia, ensuring a comprehensive overview of the early symptomatic stages. Specifically, we seek to summarize the distribution, characteristics, clinical application scenarios, accuracy metrics of these investigated AD biomarkers compared with reference standard biomarkers (Aβ-PET or CSF tests). This approach will facilitate the identification of research gaps and serve as a critical foundation for future, more focused systematic reviews or meta-analyses.

## 2 Methods

### 2.1 Protocol and registration

The scoping review followed the Joanna Briggs Institute methodological framework and was reported according to the Preferred Reporting Items for Systematic Reviews and Meta-Analyses extension for Scoping Reviews (PRISMA-ScR) [15,16]. The protocol was registered on the International Platform of Registered Systematic Review and Meta-Analysis Protocols (INPLASY) (registration number: 202,460,069, doi: 10.37766/inplasy2024.6.0069).

### 2.2 Identifying relevant studies

A literature search for studies on the early stages of AD continuum (Stages 1–4 or equivalent as defined in the National Institute on Aging – Alzheimer's Association (NIA-AA) Research Framework for Alzheimer's Disease) in the Chinese population was conducted to identify articles published from January 1, 2013 to December 31, 2023 [5,[17], [18], [19], [20]]. Four English-language databases (PubMed, EMBASE, Cochrane Library, and Web of Science) and three Chinese databases (CNKI, Wanfang, and CQVIP) were searched. For Chinese-language articles, only those published in Chinese Core Journals were included. To capture recent advances in AD biomarker research in China, a supplementary search was performed in the four aforementioned English databases for studies published between January 1, 2024 and April 30, 2025. Detailed search strategies are provided in the supplementary materials.

### 2.3 Eligibility criteria

All studies meeting the following criteria were included in this review:

1) Participants at the early stages of AD continuum based on established clinical criteria, which include preclinical AD, MCI, and mild dementia corresponding to NIA-AA Stage 1 to 4. Acceptable diagnostic criteria include NIA-AA 2018 [17], AA 2024, AAMI, International Working Group (IWG) 2 Criteria for Alzheimer's Disease Diagnosis (IWG-2) (2014) [21], Petersen criteria, or clinician assessments using various measurements. To account for historical variations in staging specificity, studies that included symptomatic AD or AD dementia populations were also eligible if their enrollment criteria permitted the inclusion of participants with MCI-AD or mild AD dementia. 2) Availability of data on Chinese participants with preclinical AD and eAD. Studies were eligible if they provided independent or subgroup data specifically for Chinese populations, including those derived from international multicenter trials (For studies involving cohorts from multiple countries, only the data relevant to the Chinese participants were extracted and synthesized). 3) Relevance to neuroimaging or fluid biomarkers for AD. Fluid Biomarkers were defined as diagnostic indicators derived from blood, CSF, and other biofluids such as saliva, urine, or feces. Neuroimaging Biomarkers were defined as imaging-based indicators used to detect AD-related brain changes. These are broadly categorized into structural imaging (e.g., sMRI for atrophy mapping), functional/metabolic imaging (e.g., fMRI or FDG-PET for neural activity and glucose metabolism), and molecular imaging (e.g., Aβ/Tau-PET for visualizing pathological protein aggregates in vivo).

The exclusion criteria were: 1) MCI or dementia caused by non-Alzheimer’s etiologies (e.g., stroke, type 2 diabetes, Parkinson’s disease, Lewy body dementia, frontotemporal dementia); 2) Patients with only moderate to severe AD dementia; 3) Studies on autosomal dominant AD or early-onset AD (onset age < 65 years); 4) Studies investigating AD treatments, including pharmacological and non-pharmacological interventions aimed at improving prognosis or alleviating symptoms; 5) Studies reporting methodological innovations or updates in fluid biomarker detection; 6) Correlation studies without diagnostic accuracy evaluations of the studied biomarkers or tools; 7) Case reports, study protocols, editorials, commentaries, opinion papers, reviews (including systematic reviews), consensus, guidelines; 8) Conference abstracts published on or before 2021; 9) Non-clinical studies, including fundamental research on molecular mechanisms conducted on animals, cells, or organoids; 10) [Supplementary search only] Clinically diagnosed AD without evidence of amyloid positivity.

### 2.4 Selection of evidence

The research results were imported and managed using EPPI Reviewer 6 (https://eppi.ioe.ac.uk/eppireviewer-web/home), with removal of any duplicate entries. EPPI-Reviewer, developed by the EPPI-Centre at the University College London, is a recommended web-based tool to support evidence synthesis [22]. Two independent researchers initially screened all titles/abstracts and then the full manuscripts to determine whether the study met the eligibility criteria. Pilot tests for both titles/abstracts and full-text screening were adopted, and the screening process started when 85% or higher agreement was achieved. Any disagreement was discussed with a third reviewer until a consensus was reached. A fourth reviewer acted as quality control during the whole evidence screening and selection process.

### 2.5 Data extraction and analysis

Two researchers independently collected data using a standardized data sheet (for detailed items, please refer to supplementary materials). Disagreements were resolved by a third researcher.

Data extraction included the following: basic information of each study, characteristics of the biomarkers investigated, their application scenarios, and metrics of diagnostic accuracy (including area under the curve [AUC], sensitivity, specificity, negative predictive value [NPV], and positive predictive value [PPV] compared with reference biomarkers). Meanwhile, considering that the internationally recognized standard for a definitive diagnosis of AD requires biomarker confirmation [17], we specifically focused on amyloid-positive cohorts (biomarker-confirmed AD), which refers to a biologically defined diagnosis requiring evidence of AD pathophysiology (e.g. amyloid positivity confirmed via CSF or PET) in addition to the clinical diagnosis. For these studies, as AUC>0.8 generally represents a strong discriminative ability in diagnostic tests [20], the investigated biomarkers with AUC>0.8 were extracted and analyzed from the perspectives of detailed features and clinical accuracy in the studies involving biomarker-confirmed AD patients. Similarly, biomarkers for distinguishing amyloid status with AUC>0.8 were also analyzed in the same way. In studies that incorporate machine learning algorithms, we extracted the optimal feature combination, as these algorithms typically generate numerous candidate combinations during feature selection. In this research, predefined data items were summarized using numbers and percentages through narrative synthesis.

## 3 Results

### 3.1 Search and selection

**Fig. 1:**
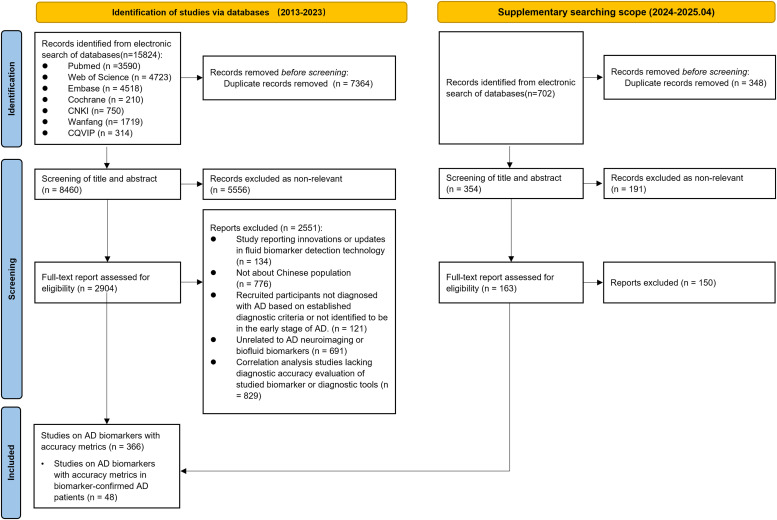
The Preferred Reporting Items for Systematic Reviews and Meta-Analyses (PRISMA) flowchart.

The primary literature search, conducted between January 1 and 11, 2024, yielded 15,824 records. After removing duplicates (7364), 8460 (53.5%) records were available for title and abstract screening. Finally, 2904 (18.4%) articles were evaluated based on the full text, and 353 articles were selected for analysis. A supplementary search was performed on May 6, 2025, to retrieve additional evidence published between January 01, 2024 and April 30, 2025, yielding 13 additional studies. In total, 366 articles were included, all of which were clinical studies on AD fluid or imaging biomarkers with accurate data from preclinical AD and eAD participants (refer to the supplementary materials for details of the included studies). Among them, we identified 48 studies investigating biomarker performance in biomarker-confirmed AD patients. The PRISMA flow diagram is shown in Fig. 1 and Figure S1, and the PRISMA-ScR checklist is provided in the supplementary materials.

### 3.2 Publication trend

**Fig. 2:**
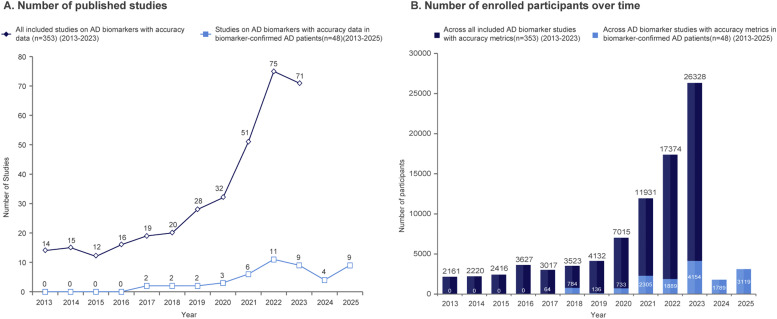
Trends in AD biomarker studies reporting accuracy metrics in the Chinese population at the early stages of AD continuum (2013–2025).

Between 2013 and 2023, clinical research on fluid and neuroimaging biomarkers in the Chinese population at the early stages of the AD continuum showed a steady upward trend, with a more pronounced increase after 2020. The total number of AD biomarker studies and enrolled participants increased rapidly, primarily driven by studies involving clinically diagnosed AD patients. Notably, despite the limited number, AD biomarker studies in biomarker-confirmed patients have risen significantly over the past five years, indicating a growing recognition of biomarker-based AD diagnosis in China (Fig. 2).

(A) Annual number of published studies on fluid or imaging biomarkers with diagnostic accuracy metrics; (B) Number of participants enrolled in all studies (2013–2023) and in studies involving biomarker-confirmed AD participants (2013–2025). Fig. 2S.

### 3.3 Source of the study population

The study populations across all included studies were derived from diverse sources: 227 studies (62.0%) recruited participants from single institutions, 63 studies (17.2%) utilized data from cohorts, and 35 studies (9.6%) involved multiple hospitals. Among the included studies, 31 AD or aging cohorts in China were identified, among which 13 cohorts used in two or more publications, with the Sino Longitudinal Study on Cognitive Decline (SILCODE) cohort generating the highest number of publications (n = 11), followed sequentially by the China Longitudinal Ageing Study (CLAS) (n = 6), the Beijing Aging Brain Rejuvenation Initiative (BABRI) (n = 5), and the Shanghai Aging Study (SAS) (n = 5) (Table S2).

### 3.4 Characteristics of investigated biomarkers

In 48 articles involving biomarker-confirmed AD patients, a total of 86 AD biomarkers with very strong discriminative ability (AUC>0.8) were identified. The summary and citations of each study are shown in Supplementary Table S1.

We found that the investigated biomarkers were employed in multiple application scenarios. Seventy biomarkers (70/86, 81.4%) were investigated in clinical scenarios for distinguishing AD populations from different control groups, with accuracy metrics reported for diagnosis (50/70, 71.4%), differential diagnosis vs other neurocognitive disorders (13/70, 18.6%), and clinical staging (7/70, 10.0%) in thirty-two studies. Additionally, 16 biomarkers (16/86, 18.6%) were recommended in nine studies assessing their concordance with reference standard biomarkers (Aβ- PET or CSF tests) for amyloid status classification regardless of the presence of AD symptoms.

### 3.5 Diagnostic biomarkers

#### 3.5.1 Characteristics of diagnostic biomarkers

Among these various clinical application scenarios, the biomarkers were more frequently used for diagnosis, with 50 biomarkers from 27 studies achieving very strong discriminative ability in distinguishing AD patients from healthy controls (AUC>0.8).

Of these 50 diagnostic biomarkers, fluid biomarkers constituted the majority (82.0%, 41/50), derived from blood (54.0%, 27/50; 4 serum, 23 plasma), CSF (22.0%, 11/50), gut microbiota (4.0%, 2/50), and urine (2.0%, 1/50). Among the fluid biomarkers, two were combined with machine learning models. One of these biomarkers was a combination of six plasma digital spectral biomarkers, and the other is a combination of plasma 21 proteins, including multiple AD pathway-related proteins.

The remainder were primarily neuroimaging biomarkers (16.0%, 8/50), and one multimodal biomarker integrating both imaging and plasma-derived variables (2.0%, 1/50). Three of them incorporated machine learning techniques: (1) a multimodal biomarker integrating fluid biomarkers (plasma GFAP, NfL, and their ratios), tau PET imaging, and demographic variables; (2) a neuroimaging biomarker combining multi-region features from structural MRI [sMRI]; and (3) a neuroimaging biomarker integrating optimally combined Regions of Interest (ROIs) from [¹¹C] Pittsburgh Compound-B PET (PiB-PET).

**Fig. 3:**
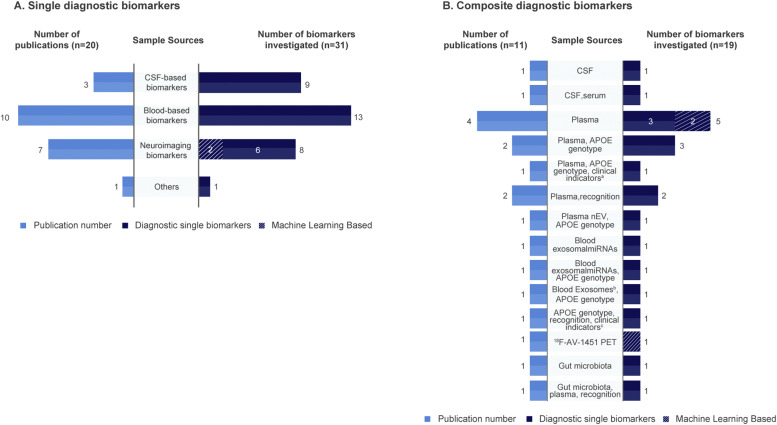
Distribution of studies and diagnostic biomarkers by different sample sources. (A) Single diagnostic biomarkers; (B) Composite diagnostic biomarkers.

When categorized by composition, 62.0% (31/50) of all diagnostic biomarkers were single biomarkers, and the remainder were composite. The single biomarkers were predominantly blood-based (41.9%, 13/31; 1 serum, 12 plasma), and CSF-based (29.0%, 9/31), and only eight (25.8%) were single neuroimaging biomarkers—defined as those derived from a single neuroimaging modality (e.g., sMRI or functional MRI [fMRI]), even if multiple parameters within that modality were integrated (Fig. 3A).

a. Clinical indicators included age, sex, years of education; b. Blood neuro-exosomal synaptic. Abbreviations: miRNAs, microRNAs. Note. Only biomarkers with very strong discriminative ability (AUC>0.8) are included.

Additionally, there were 19 composite diagnostic biomarkers (19/50, 38.0%), derived from various sample sources and mostly reported in only one study (Fig. 3B).

#### 3.5.2 Clinical applications of diagnostic biomarkers

The biomarkers investigated were used to distinguish AD from healthy controls across different stages in clinical studies. The most common application is distinguishing AD dementia patients (ADD) from healthy controls (47.1%), followed by distinguishing early symptomatic AD patients (MCI-AD and mild AD dementia) from healthy controls (21.4%), and distinguishing MCI-AD alone (11.4%). The proportion of biomarkers used to identify a preclinical AD population (identifying AD pathology among asymptomatic individuals) is 15.7%, and only a small proportion were used to identify both preclinical and MCI-AD (4.3%) from healthy controls.

Among all the diagnostic biomarkers, only six biomarkers demonstrated good discriminative abilities for diagnosing AD in individuals with early symptoms and were validated in more than one study: plasma p‐tau217, plasma p-tau181, plasma GFAP, plasma Aβ42 and CSF Aβ42, and CSF t-tau. All of them are single biofluid-based biomarkers.

#### 3.5.3 Diagnostic accuracy for biomarker-confirmed MCI-AD

**Fig. 4:**
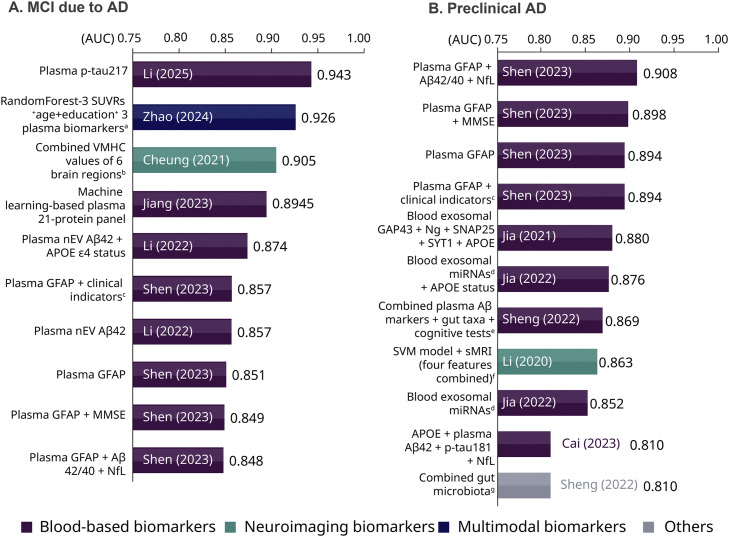
AUC of diagnostic biomarkers distinguishing biomarker-confirmed MCI-AD and preclinical AD participants from healthy controls. **(**A). MCI-AD; (B) preclinical AD.

For diagnosing AD at the MCI stage, distinguishing MCI-AD participants with amyloid positivity from cognitively unimpaired controls, we found that eight biomarkers demonstrated very strong discriminative ability (AUC>0.8). The biomarkers were plotted according to the AUC values (Fig. 4A) and collect corresponding accuracy metrics such as sensitivity, specificity, NPV, PPV, and study information (Table S3). None of the biomarkers met the criteria of confirmatory test (sensitivity≥90%, specificity≥90%) recommended by the Global CEO Initiative on AD (CEOi) [23].

a. Three plasma biomarkers included NfL, GFAP, GFAP/NfL; b. The biomarker combined VMHC values of inferior frontal operculum, Rolandic operculum, supplementary motor area, inferior frontal orbital gyrus, and gyrus rectus; c. Clinical indicators included age, sex, years of education, *APOE* ε4 allele. d. A panel of six miRNAs (upregulated: miR-29c-5p, miR-143–3p, miR-335–5p, and miR-485–5p; downregulated: miR-138–5p and miR-342–3p); e. Plasma Aβ markers combined plasma Aβ40, Aβ42, and Aβ42/Aβ40; combined gut taxa included taxa 1, taxa 2, and taxa 3 (taxa 1, combined phylum Bacteroidetes, class Bacteroidia, and order Bacteroidales; taxa 2, combined phylum Firmicutes, class Clostridia, order Clostridiales, family Lachnospiraceae, family Ruminococcaceae, and genus Faecalibacterium; taxa 3, combined class Deltaproteobacteria, order Desulfovibrionales, family Desulfovibrionaceae, and genus Bilophila). Cognitive tests included MoCA-B, AVLT-long delayed recall, AVLT-Recognition, STT-A (the Shape Trails Test Part A), STT-B (the Shape Trails Test Part B), AFT (the Animal Fluency Test), and BNT (the 30-item Boston Naming Test). f. Four sMRI features were large zone high-gray-level emphasis (LZHGE) feature of the left posterior cingulate gyrus on sMRI combined with three stable high-frequency features on sMRI, including LZHGE feature of the right posterior cingulate gyrus, the variance feature of the left superior parietal gyrus and the coarseness feature of the left posterior cingulate gyrus; g. Combined gut microbiota included taxa 1, taxa 2, and taxa 3. Taxa 1, combined phylum Bacteroidetes, class Bacteroidia, and order Bacteroidales; Taxa 2, combined phylum Firmicutes, class Clostridia, order Clostridiales, family Lachnospiraceae, family Ruminococcaceae, and genus Faecalibacterium; Taxa 3, combined class Deltaproteobacteria, order Desulfovibrionales, family Desulfovibrionaceae, and genus Bilophila. **Abbreviations: MCI-AD,** mild cognitive impairment due to AD; MMSE, Mini-Mental State Examination scores; MoCA, Montreal Cognitive Assessment; AVLT N5 auditory verbal learning test-delayed memory; AVLT N7 auditory verbal learning test-recognition; p-tau, phosphorylated tau; SUVR, standardized uptake value ratio; VMHC, voxel-mirrored homotopic connectivity; nEV, neuronal-derived extracellular vesicle; GFAP, Glial Fibrillary Acidic Protein; Aβ, amyloid β-protein; NfL, neurofilament light chain; t-tau, total tau; CSF, cerebrospinal fluid; AβPP, the platelet amyloid-protein precursor; NTP, neuronal thread protein, NAA/Cr, N-acetylaspartate /creatine; Ng, neurogranin; SYT1, synaptotagmin 1.

##### 3.5.3.1 Plasma biomarkers

Plasma p-tau217 tested by SIMOA achieved the highest AUC value of 0.943. However, other accuracy metrics such as sensitivity and specificity were not reported. The study also found that the reference range for plasma p-tau217 in the Chinese population is 0.006–0.47 pg/mL, lower than the 0.4–0.63 pg/mL reported in Western cohorts.

Another plasma 21-protein panel biomarker was developed using machine learning. Designed to include multiple proteins from key AD pathways, it demonstrated high efficacy with an AUC of 0.8945 for identifying MCI due to AD.

##### 3.5.3.2 Neuroimaging biomarkers

One neuroimaging biomarker was identified [24,25]. It utilized voxel-mirrored homotopic connectivity (VMHC) in resting-state functional MRI. Through combining the VMHC values at six relevant brain regions (inferior frontal operculum, Rolandic operculum, supplementary motor area, inferior frontal orbital gyrus, gyrus rectus, and superior frontal orbital gyrus), the diagnostic capabilities achieved an AUC of 0.905 with 83% accuracy (sensitivity 88%, specificity 83%).

##### 3.5.3.3 Multimodal biomarkers

Another biomarker incorporated machine learning, using a random forest model to integrate eight variables (left amygdala standardized uptake value ratio [SUVR], right amygdala SUVR, left entorhinal cortex SUVR from tau PET, age, education, plasma NfL, plasma GFAP, plasma GFAP/NfL), and achieved an AUC of 0.926 (sensitivity 93.9%, specificity 85.6%), which has met the criteria of high-specificity triaging test recommended by CEOi (sensitivity≥90%, specificity≥85%) [23].

#### 3.5.4 Detection of preclinical AD

Eleven biomarkers were found for identifying preclinical AD (identifying AD pathology in asymptomatic individuals) with very strong discriminative ability (AUC>0.8) from amyloid-negative individuals (Fig. 4B, Table S4). None of the biomarkers had met the criteria of confirmatory test (sensitivity≥90%, specificity≥90%) [23].

##### 3.5.4.1 Plasma biomarkers

Currently, four plasma GFAP-related biomarkers from one study demonstrated relatively strong discriminative ability to identify preclinical AD [14]. Among them, only a single biomarker (plasma GFAP) provided a complete set of diagnostic performance metrics — including sensitivity (88.20%), specificity (83.60%), PPV (73.2%), and NPV (93.9%) — at a cut-off value of 68.59 ng/mL.

In addition, two biomarkers derived from blood exosomes were reported by the same research team, both achieving high and comparable AUC values of 0.85 - 0.88 [12,26]. Neuro-exosomal synaptic proteins (GAP43, neurogranin, SNAP25, synaptotagmin 1) and a panel of exosomal miRNAs (miR-29c-5p, miR-143–3p, miR-335–5p, miR-485–5p, miR-138–5p and miR-342–3p) with or without *APOE* genotype status demonstrated the ability to detect preclinical AD five to seven years before the onset of cognitive impairment.

##### 3.5.4.2 Neuroimaging biomarkers

Only one neuroimaging biomarker was identified for preclinical AD, which combined machine learning approach and sMRI features. It integrated three stable high-frequency sMRI features along with feature 6486 using a support vector machine (SVM) model, achieving an AUC of 0.863 [27].

### 3.6 Biomarkers for distinguishing amyloid status

Nine studies used PET or CSF results as reference values to investigate the ability of biomarkers and their combinations to recognize amyloid positivity. All these studies were published between 2023 and 2025. A total of 16 biomarkers were recommended in these studies (Table S5).

**Fig. 5:**
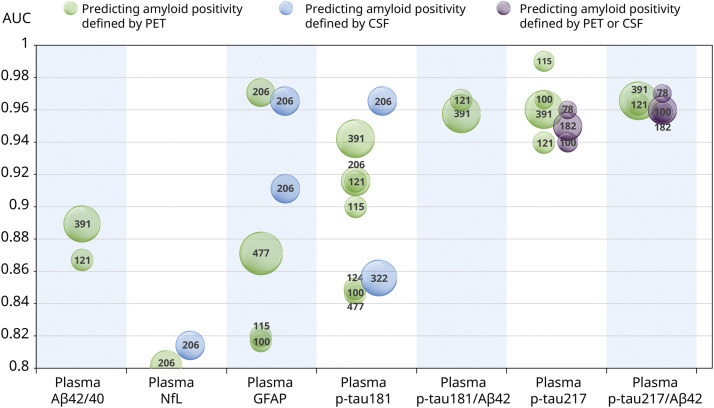
Biomarkers for distinguishing amyloid-positive from amyloid-negative individuals tested with ≥2 cohorts.

Note. Validation cohorts are depicted as circles whose area is proportional to sample size. Color coding corresponds to the methodology employed for amyloid positivity classification within each cohort.

Among the biomarkers identified, seven biomarkers were validated across multiple cohorts within the Chinese population. These biomarkers were all single plasma biomarkers and demonstrated robust diagnostic performance, with AUC values consistently exceeding 0.8 when compared against gold-standard measures (amyloid positivity by PET or CSF). The amount of validation evidence varied among biomarkers, with p-tau181/Aβ42 being supported by the largest body of evidence (9 cohorts), followed by p-tau217 (7 cohorts) and p-tau217/Aβ42 (5 cohorts). The strength of validation is illustrated by both the number of supporting cohorts (represented by circle count) and sample sizes (represented by circle size) in Fig. 5 [[28], [29], [30]]. These three biomarkers all achieved an AUC over 0.9 for predicting amyloid positivity by CSF or PET across all validation cohorts. They also met the minimum acceptable criteria in the Chinese population for clinical use as confirmatory tests recommended by CEOi, with a sensitivity and specificity of ≥90%, as well as a PPV and NPV of ≥90%, when the Aβ prevalence is around 50%.

## 4 Discussion

### 4.1 Biomarker-confirmed AD research in China

According to the AA 2024 framework, AD constitutes a continuum defined by biological characteristics. The disease begins with the appearance of changes in the brain associated with the disease processes in asymptomatic individuals, and progresses through stages of increasing levels of pathology, eventually leading to the appearance and progression of clinical symptoms. In contrast, the IWG defines individuals who are biomarker-positive but cognitively unimpaired as “asymptomatic at risk of AD”. However, both AA and IWG recognize that accurate biomarkers are key for supporting the early identification and diagnosis of AD in clinical practice, in conjunction with a clinical evaluation. Although most studies are still conducted in clinically diagnosed AD participants, the number of studies validating the accuracy and performance of novel AD biomarkers (vs Aβ-PET or CSF), including studies in early symptomatic and even asymptomatic individuals, has significantly increased in the Chinese population, particularly in the last five years. This trend reflects the rapid development of biomarker-related research in the Chinese population, alongside growing academic interest in the clinical utility of novel biomarkers compared to established PET or CSF biomarkers. The predominant focus on blood-based biomarkers in Chinese studies aligns with global research priorities [31,32]. However, it is evident that studies on multimodal biomarkers remain relatively scarce compared to those on single biomarkers. Beyond scientific considerations, factors such as economic cost and practical accessibility may also contribute to the relatively limited investigation into multi-source composite imaging biomarkers for diagnostic purposes.

### 4.2 AD/aging cohorts in China

In this review, we found that 63 studies involved participants from Chinese AD or aging cohorts. Cohorts such as SILCODE, CLAS, BABRI, and SAS were reported in at least two studies. These cohorts adopted uniform enrollment criteria across multiple recruiting centers, ensuring high-quality and homogeneous participant data within each cohort. This enabled the establishment of a comprehensive dataset with complete clinical information, extensive biomarker profiles, and longitudinal follow-up data. These cohorts exhibited certain variations, including differences in regional coverage, the clinical stages of AD among enrolled individuals, and the spectrum of neurodegenerative diseases studied, among others. Such rich and well-structured datasets provide a powerful foundation for supporting high-quality Alzheimer's research in China, comparable to resources like the Alzheimer's Disease Neuroimaging Initiative (ADNI) [33], [34].

### 4.3 Diagnostic biomarkers for MCI-AD

Our study reveals that the most extensively researched application of AD biomarkers currently is in diagnosis. Among these studies, nearly half (45.8%) of the studies employed biomarkers for diagnosing AD in individuals at the dementia stages and only 20.8% were used to diagnose AD in individuals in the MCI stage. This may be due to the fact that early symptoms of AD are often subtle and easily overlooked, leading to delayed medical consultation. As a result, the majority of patients seek care only at the dementia stage. Additionally, the reviewed studies spanned a considerable period, with diagnosis of AD in earlier studies relying on overt dementia symptoms as well, while mild dementia was not separately identified as a distinct category [[35], [36], [37]].

Therefore, there remains a critical unmet need for validated biomarkers to confirm AD pathology in individuals at the MCI stage [38]. This stage represents the primary therapeutic window for recent AD disease-modifying therapies, including two China-approved amyloid-targeting antibodies, lecanemab and donanemab, which has shown significant efficacy in attenuating cognitive decline within the MCI population [39,40]. Nevertheless, we found that several biomarkers were studied for diagnosing Chinese patients at the MCI-AD stage with good performance, which is conducive to the early identification and early drug intervention.

Our review found that plasma p-tau217 demonstrated strong diagnostic performance for biomarker-confirmed MCI-AD patients in the Chinese population, achieving an AUC of 0.943 [41]. Previous studies have widely recognized that plasma p-tau217 has the ability to identify Aβ-PET positivity and has an accuracy comparable to that of CSF p-tau217 [29]. A meta-analysis of 30 studies showed that both the sensitivity and specificity of plasma p-tau217 in detecting Aβ protein and tau pathology in Alzheimer's disease exceeded 80% [42]. At present, Vazyme' plasma p-tau217 kit has been approved for marketing and application in China, and has been used in clinical practice to assist in the diagnosis of AD [43]. In the future, we expect more large-scale validation data on plasma p-tau217 applied to AD patients at different stages.

### 4.4 Diagnostic biomarkers for preclinical AD

The concept of preclinical AD has been officially recognized in recent years [19]. These patients are very likely to experience symptoms of cognitive decline in the future, and therefore biomarker-based screening and diagnosis are of greater significance for this particular patient group [7,17]. That said, our review still found that 15.3% of studies focused on biomarkers for identifying individuals with preclinical AD, with the majority relying on plasma biomarkers.

Regarding the preclinical AD population, a recent study demonstrated that p-tau217 exhibits high accuracy in identifying preclinical AD, despite the limited PPV low amyloid positivity [44]. Notably, there is a marked scarcity of specific studies validating p-tau217 assays within biomarker-confirmed Chinese preclinical AD cohorts. This gap is primarily attributable to the fact that many current Chinese studies group preclinical AD with MCI or symptomatic AD, making it difficult to extract independent performance data for the preclinical stage [45]. Furthermore, validating p-tau217 in this population necessitates studies that provide either baseline biomarker verification or extended clinical follow-up to confirm phenotypic conversion. Currently, such resources with comprehensive baseline data remain limited. Nevertheless, as biomarker-confirmed diagnosis becomes increasingly prevalent and ongoing longitudinal studies mature, we anticipate that more robust evidence specifically for the Chinese preclinical population will soon emerge. This review identified a publication showed that four plasma GFAP-related biomarkers were with a strong ability to discriminate preclinical AD from cognitive unimpaired individuals [14]. Other studies have shown similar diagnostic potential of GFAP in differentiating amyloid-positive from negative cognitively unimpaired individuals [46,47]. However, plasma GFAP is not a specific biomarker for AD, and its elevated concentration may also be associated with other neurodegenerative diseases, inflammatory conditions, metabolic status, etc. [48]. Meanwhile, there is accumulating evidence for the utility of GFAP in preclinical AD in the Chinese population. A previous study reported that plasma GFAP levels predicted conversion to amyloid positivity, suggesting that elevations in GFAP may occur early in the disease process [46]. Additionally, a longitudinal study showed that each one standard deviation unit (SDU) increase in log-transformed circulating GFAP levels was associated with an approximate 2.5-fold higher risk of all-cause and AD dementia over the up to 15-year follow-up period [47]. These studies indicate that elevations in plasma GFAP may occur in the early stages of the pathophysiological process of AD.

Nevertheless, studies in the Chinese population remain relatively limited. Whether more specific biomarkers, such as plasma p-tau217 or p-tau231, can be effectively applied for the identification of preclinical AD in Chinese population still requires further exploration [49].

### 4.5 Biomarkers for distinguishing amyloid status irrespective of clinical symptoms

Although evidence remains limited regarding the use of biomarkers for diagnosing AD in the Chinese population at early disease stages, p-tau181/Aβ42, p-tau217, and p-tau217/Aβ42 have demonstrated a robust and consistent ability to identify amyloid positive pathology across multiple Chinese cohorts. Evidence suggests that the performance of these three biomarkers in the Chinese population meets the high-performance standards recommended by the CEOi for use as confirmatory tests. These findings indicate a strong potential of these three biomarkers for diagnosing AD in clinical practice in the Chinese population with cognitive complaints [28,30,41,50,51].

The Lumipulse G p-tau217/Aβ42 ratio is the first in vitro diagnostic reagent approved by the FDA to assist in the diagnosis of AD through blood tests. The FDA evaluated the data from a multicenter clinical study covering 499 patients with impaired cognitive function. Taking amyloid positivity determined by PET or CSF as references, it exhibited high diagnostic accuracy for assessing amyloid positivity [52]. It is worth noting that, plasma p-tau217/Aβ42 had also exhibited high diagnostic accuracy for abnormal statuses of Aβ-PET and tau-PET with Aβ-PET and tau-PET as references in clinical (n = 391) and community cohorts (n = 121) in China. This performance was clinically equivalent to the performance of CSF p-tau181/Aβ42 and Aβ42/Aβ40 and higher than those of plasma p-tau217, Aβ42/Aβ40, p-tau181, and p-tau181/Aβ42 in both clinical and community cohorts [29]. The high diagnostic accuracy of plasma p-tau217/Aβ42 observed in the Chinese population confirms robust utility. In the future, after its accuracy in diagnosing AD is further confirmed in large-scale clinical practice in China, it is very likely to become another biomarker to replace the reference PET or CSF markers in the Chinese population.

Besides amyloid related biomarkers, Jiang YB et al. (2024) developed a proteomic detection method capable of simultaneously detecting 21 AD-related biomarkers in plasma. The biomarker, namely the 21-protein panel, developed by a team from Hong Kong and China, involves proteins in five key AD pathological pathways: neurodegeneration, inflammation, innate immunity, vascular function, and metabolic activity. The 21-protein panel study found that this panel has a good ability to identify Aβ-PET confirmed MCI patients in the Chinese population, demonstrating strong diagnostic potential that is superior to traditional ATN (Aβ, Tau, and Neurodegeneration) markers. The study also identified differences in the biological pathways of AD between Chinese and Hispanic populations. At the MCI-AD stage, Chinese people are mainly characterized by inflammation and congenital immune abnormalities, while Hispanic people are mainly characterized by vascular dysfunction and congenital immune abnormalities, suggesting the possibility of multi-mechanism and the need to develop race-specific interventions in the early stage of AD [53].

### 4.6 Biomarkers incorporating machine learning-based models

In this review, we identified four studies that investigated machine learning–based biomarkers for diagnosing preclinical and eAD, or for identifying amyloid positivity. Across these studies, machine learning was integrated with blood‑based, neuroimaging, or multimodal biomarkers, with or without demographic features. All demonstrated good diagnostic performance [10,25,53,54]. In fact, many studies on integrated machine learning–based biomarkers was identified during screening, but most of them have not been validated in biomarker-confirmed AD populations[[55], [56], [57]]. With the rapid advancement of machine learning and digital technology, the integration of large language models, multimodal data fusion, and other cutting-edge technologies into clinical diagnosis and treatment has become increasingly valuable. Machine learning-assisted biomarkers are expected to achieve ultra-early screening and risk prediction of AD with smaller sample sizes and more accurate parameters, and might be embedded in mobile applications (apps) for more extensive clinical applications [58,59].

## 5 Limitations

During the process of conducting a scoping review, although we ensured that each study had clear diagnostic criteria based on inclusion and exclusion, there may be differences in the staging of patients and the collection and detection methods of biomarkers in each study. Also, some biomarkers only reported AUC values, lacking data on sensitivity and specificity. Therefore, summaries based on AUC can only be used as references for actual clinical applications. Furthermore, this study primarily focuses on clinical research related to the diagnosis of AD based on biomarkers. Some studies that tested biomarkers in large cohorts of patients clinically diagnosed with Alzheimer's disease, despite demonstrating promising diagnostic performance and clinical application potential, were not included. This review is limited to focusing on fluid and neuroimaging biomarkers. Other types of AD-related biomarkers, such as retinal imaging biomarkers and digital phenotypes, are currently under investigation and warrant further attention.

To comprehensively map the landscape of AD biomarker research, this scoping review included a large number of studies with substantial heterogeneity. A formal risk of bias assessment and statistical synthesis were not performed. However, future work may leverage the gaps and priorities identified herein to perform targeted systematic reviews or meta‑analyses focusing on specific biomarkers or clinical application scenarios.

### 5.1 Implications for future studies

In China, many AD or aging cohorts have been established. A future direction would be to incorporate these cohorts into a national, more representative cohort with comprehensive cross-sectional and longitudinal patient data, similar to the ADNI database. Such efforts would help reduce regional differences in representativeness, integrate data from multiple visits of the same patients across different hospitals, and support tracking of the disease progression in early-stage AD patients within real-world settings, as well as assessing the possible impact of different risk factors and intervention measures.

Research on AD biomarkers in China is in line with international research trends. Several blood biomarkers have been verified to be capable of recognizing amyloid status in the AD population in China, meeting the diagnostic criteria. Subsequent studies can further verify the ability of these blood biomarkers to diagnose AD patients at different clinical stages in real clinical settings, based on the Chinese cohorts. Moreover, more research is needed on these biomarkers for the staging of AD patients and for predicting disease onset and progression, as well as biomarkers involved in multiple biological pathways such as neuroinflammation and vascular health.

Currently, machine learning algorithms have demonstrated that neuroimaging markers based on a single sample source and multi-sample composite markers have excellent capabilities in diagnosing the early stages of the AD continuum. With the continuous development of artificial intelligence (AI) technology, it is expected that more machine learning-assisted biomarkers will demonstrate their application potential in diverse clinical scenarios in the future.

## 6 Conclusions

This scoping review systematically synthesizes a decade of research on fluid and imaging biomarkers for AD in China. We found that a growing number of studies on biomarkers have been conducted in AD patients confirmed by reference biomarkers (PET or CSF), especially over the past five years. In the Chinese population, the accuracy of plasma p-tau217-based biomarkers in diagnosing AD in the MCI and dementia stages has been verified, with plasma p-tau217, p-tau181/Aβ42, and p-tau217/Aβ42 reaching the diagnostic level recommended by CEOi. Although the number of imaging markers studied in the biomarker-confirmed AD population was small, some single-source markers, such as the features of sMRI, also demonstrated excellent diagnostic performance after integrating machine learning algorithms. We anticipate a surge in research on biomarkers in China, focusing on: robust validation studies, markers from diverse biological pathways, applications for staging and prediction, and AI-assisted discovery. This expansion will ultimately contribute to improved patient stratification and management strategies for AD.

## Funding

This scoping review was funded by Novo Nordisk (China) Pharmaceuticals Co., Ltd.

## Declaration of generative AI and AI-assisted technologies

We confirm that no generative AI or AI-assisted technologies were used in the writing, or the creation of figures, images, and artwork

## Ethics statement

This article is a scoping review based on previously published studies and does not involve any new investigations on human participants or the collection of primary data. Therefore, ethics approval and informed consent are not required.

## Registration number

The protocol was registered on the International Platform of Registered Systematic Review and Meta-Analysis Protocols (INPLASY) (registration number: 202,460,069, doi: 10.37766/inplasy2024.6.0069).

## Data statement

The data supporting the findings of this study are available within the article and its Supplementary Materials. The full extracted dataset is available from the corresponding author upon reasonable request.

## CRediT authorship contribution statement

**Guoping Peng:** Conceptualization, Methodology, Supervision, Writing – review & editing. **Yan Yang:** Investigation, Data curation, Formal analysis, Writing – original draft. **Ying Wang:** Methodology, Data curation, Formal analysis, Writing – original draft. **Sagar Anil Chandekar:** Formal analysis, Writing – review & editing. **Jintai Yu:** Conceptualization, Methodology, Supervision, Writing – review & editing.

## Declaration of competing interest

The authors declare the following financial interests/personal relationships which may be considered as potential competing interests:

Yan Yang, Ying Wang reports financial support was provided by Novo Nordisk China Pharmaceuticals Co., Ltd. If there are other authors, they declare that they have no known competing financial interests or personal relationships that could have appeared to influence the work reported in this paper.

## Appendix Supplementary materials

Image, application 1

Image, application 2

Image, application 3
