# Supplementary material for: Biomarkers in preclinical and early Alzheimer’s disease in China: a scoping review
Source: J Prev Alzheimers Dis. 2026 May 19;13(7):100599. doi: 10.1016/j.tjpad.2026.100599 (PMC13213292; doi:10.1016/j.tjpad.2026.100599)
Supplement: Supplementary file 3 [file mmc3.docx]

**Biomarkers in preclinical and early Alzheimer’s disease in China: a scoping review**

**Supplementary Materials**

**Table of Contents**

[Supplementary File 1 Search strategies 2](#_Toc224686776)

[Figure S1 PRISMA flowchart of studies identified for inclusion in the supplementary search (20240101-20250430) 3](#_Toc224686777)

[Figure S2. Distribution of the investigated biomarkers with good performance and related publications across clinical application scenarios. 4](#_Toc224686778)

[Table S1. Summary of studies in biomarker-confirmed AD with AUC>0.8. 5](#_Toc224686779)

[Table S2. Cohorts generating ≥2 studies reporting accuracy metrics on fluid or imaging biomarkers for early stages of AD 14](#_Toc224686780)

[Table S3. Biomarkers with AUC>0.8 for diagnosing biomarker-confirmed MCI-AD 16](#_Toc224686781)

[Table S4. Biomarkers for diagnosing preclinical AD with AUC>0.8 20](#_Toc224686782)

[Table S5. The recommended biomarkers for distinguishing amyloid-positive from amyloid-negative individuals 24](#_Toc224686783)

Supplementary File 1 Search strategies

The keywords and MeSH terms used for Alzheimer’s disease were “Alzheimer* ” and “Alzheimer disease”. To further confine the research population to the early stages of the AD continuum, the following keywords are included: early, mild cognitive impairment, MCI, mild neurocognitive disorder, aMCI, amnestic mild cognitive impairment, early AD, mild AD, prodromal AD, pre-dementia, early dementia, mild dementia, mild cognitive dysfunction, mild cognitive decline, preclinical AD, subjective cognitive decline, subjective cognitive impairment, subtle cognitive impairment, possible AD, probable AD. Keywords including China, Chinese, People's Republic of China, Republic of China, Hong Kong, Hongkong, Taiwan, Macau, and Macao were added as the scoping review will focus on the early stages of AD continuum in the Chinese population. Non-human studies and articles focusing on AD treatment using the keywords treatment, therapy, Huperzine, medicine, traditional Chinese medicine, pharmacy, acupuncture, lecanemab, animal, cellular, and in vitro in the title and mouse, mice, rat, rats, rabbit, and rabbit in the title/abstract were excluded. Keywords were supplemented with appropriate thesaurus terms according to the databases searched. For the supplementary search, we retained the primary search strategy and added keywords/MeSH terms (biomarker, AUC, sensitivity, specificity, PPV, NPV, accuracy) to focus on studies reporting effectiveness data for AD biomarkers.

**
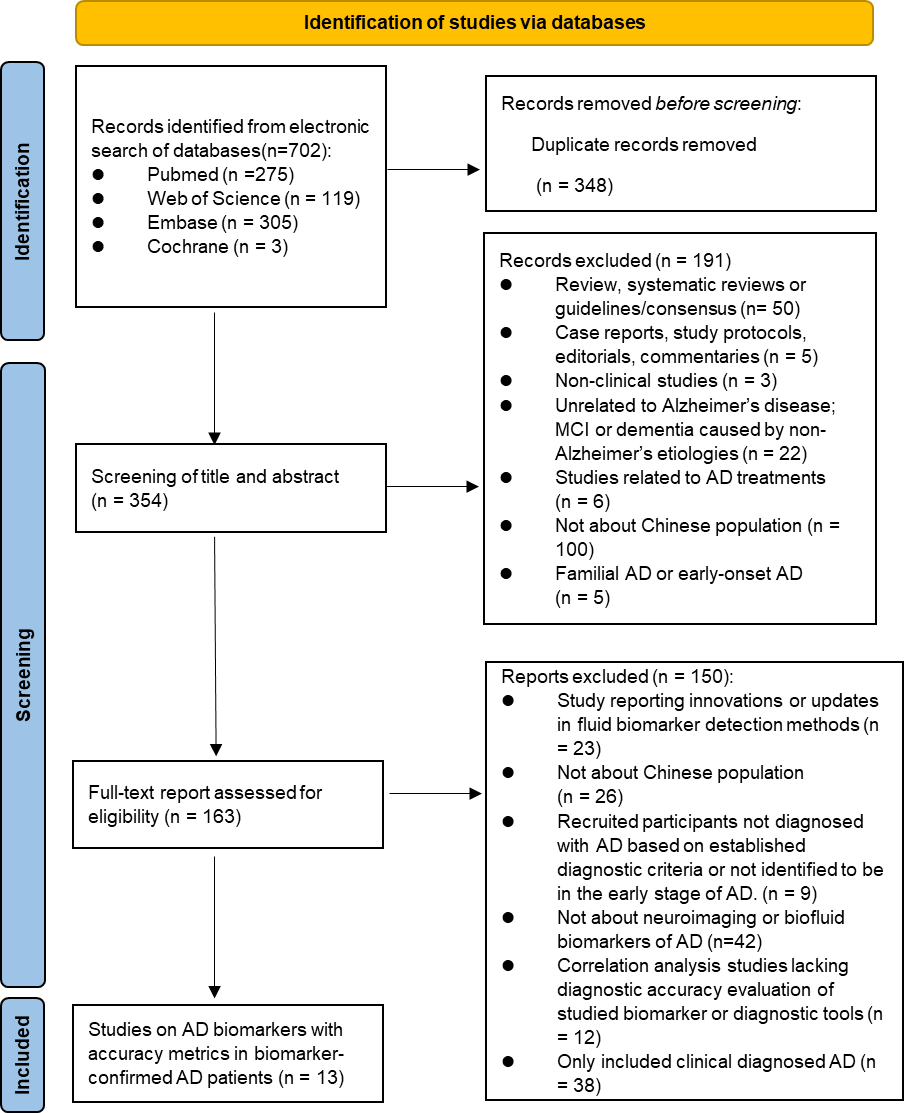
**

Figure S1 PRISMA flowchart of studies identified for inclusion in the supplementary search (20240101-20250430)


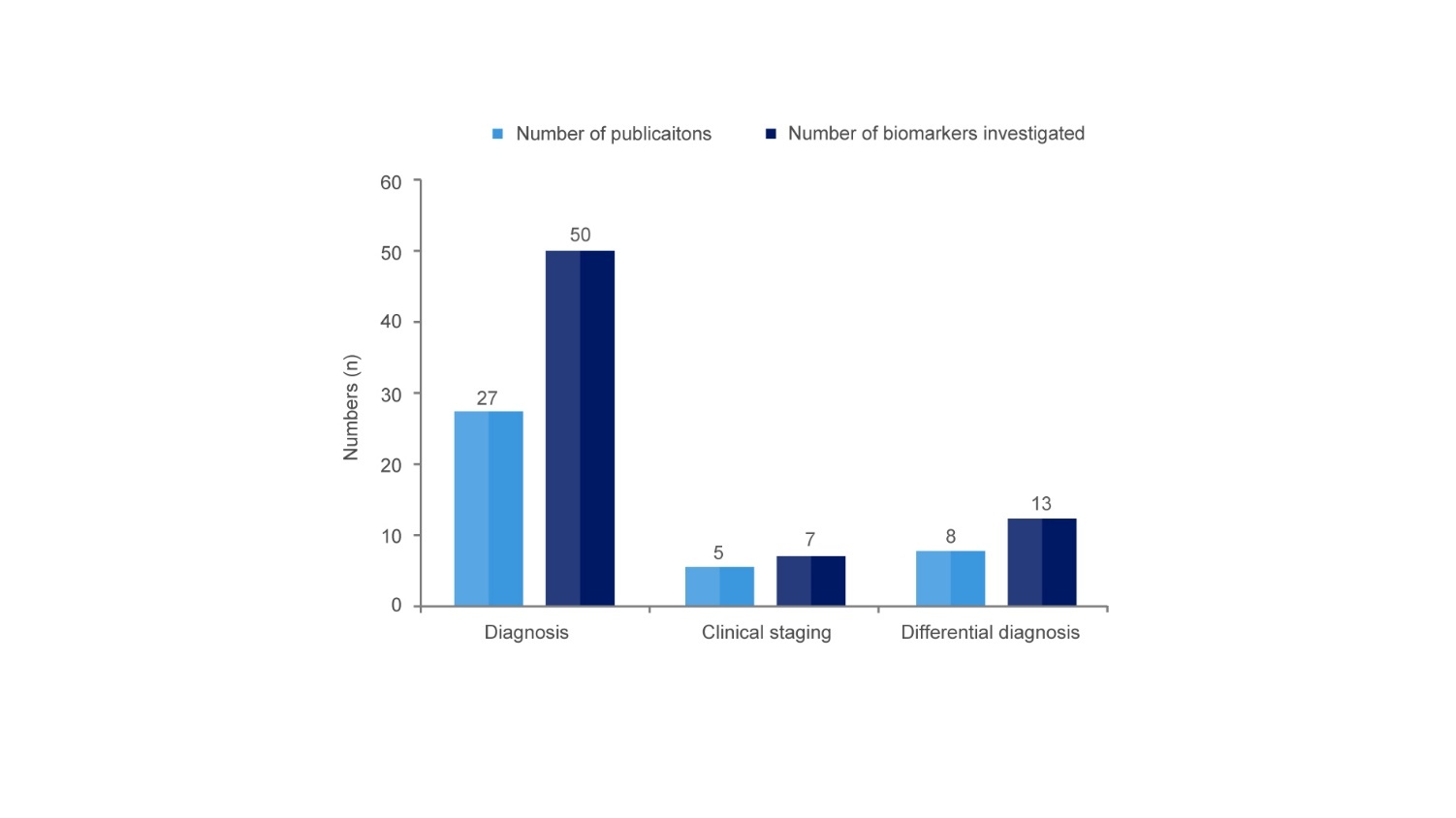


Figure S2. Distribution of the investigated biomarkers with good performance and related publications across clinical application scenarios.

Note. Each biomarker is counted only once per scenario, and only those with strong discriminative ability (AUC>0.8) are included.

Table S1. Summary of studies in biomarker-confirmed AD with AUC>0.8.

| Included study | | Number of Participants | Investigated biomarker & diagnostic tools | Current Findings |
| --- | --- | --- | --- | --- |
| Jiao (2020) | AD | 40 | PET-PiB | Plasma Aβ42 and t-tau are biomarkers that can be used to support the diagnosis of AD, reflecting amyloid pathology and neurodegeneration, respectively. In clinical studies targeting individuals with early symptomatic AD, plasma Aβ42 and t-tau (similar to CSF t-tau) have been validated across multiple studies for their strong discriminative ability, making them effective tools for identifying AD patients compared to healthy controls. |
|  | NC | 57 |  |  |
| Sheng (2022) | CN- | 34 | [18F] florbetapir (AV-45) PET | Plasma Aβ42 and the Aβ42/Aβ40 ratio are frequently investigated as biomarkers for evaluating AD pathological changes and brain amyloidosis. Furthermore, as previously reported, the gut microbiota (fecal microbial structure) in AD patients differs significantly from that of controls; such differences in gut taxonomic composition have also been investigated as fluid biomarkers for identifying early or preclinical stages of AD. |
|  | CN+ | 32 |  |  |
| Li (2022) | Aβ− NCs | 84 | [18F] florbetapir (AV-45) PET | Neuronal-derived extracellular vesicles (nEVs), as emerging fluid biomarkers, demonstrate potential in diagnosing AD-induced cognitive impairment (such as MCI-AD) and identifying asymptomatic preclinical AD stages with high sensitivity. |
|  | Aβ+ NCs | 72 |  |  |
|  | aMCI | 45 |  |  |
|  | ADD | 45 |  |  |
| Au (2021) | control group | 95089749 | Amyloid PET Tau PET | Multimodal models incorporating clinical and radiologic (neuroimaging) data can be used to differentiate and classify subjects with early AD and controls. |
|  | early AD | 95089749 |  |  |
| Bao (2021) | SCD | 13 | Amyloid PET | Aβ positivity (e.g., detected via PET imaging) and related neuroimaging metrics (such as the standardized uptake value ratio, SUVR) can be used to evaluate and differentiate various clinical stages of AD, such as MCI |
|  | MCI | 39 |  |  |
|  | AD | 22 |  |  |
| Cai (2023) | China COAST-control-baseline | 123 | CSF Aβ42， CSF p-tau181/Aβ42 | In Chinese populations, plasma biomarkers (such as Aβ42, p-tau181, and NfL) have been investigated for identifying preclinical AD. These blood-based markers show concordance with their CSF counterparts and can be used to discriminate preclinical AD patients from healthy controls. |
|  | China COAST-PreAD | 126 |  |  |
|  | China COAST-control-Follow-up | 123 |  |  |
|  | China COAST-AD | 126 |  |  |
| Chen (2022) | Control | 37 | Amyloid PET & tau PET | N-acetylaspartate/creatine (NAA/Cr) is one of the biomarkers involved in Alzheimer's disease (AD) research. |
|  | Early AD | 16 |  |  |
|  | Late AD | 15 |  |  |
| Jia (2021) | Baseline-Preclinical AD | 160 | P-tau/Aβ42 (0.14) and T-tau/Aβ42 (0.67) | Neuro-exosomal synaptic proteins (including GAP43, neurogranin, SNAP25, and synaptotagmin 1) serve as effective biomarkers capable of predicting or detecting preclinical AD five to seven years before the onset of cognitive impairment. |
|  | Baseline-Controls | 160 |  |  |
|  | Follow-up：Preclinical AD | 160 |  |  |
|  | Follow-up：Controls | 160 |  |  |
| Lin (2019) | Amyloid PET Positive | 22 | 18F-florbetapir amyloid PET | APOE genotypes and plasma Aβ42 are important indicators included in biomarker research for early Alzheimer's disease, encompassing early dementia or mild cognitive impairment stages. Studies demonstrate that related blood-based biomarkers and their multimodal combinations can be utilized to predict or identify patients' amyloid pathology status, such as predicting the positivity of amyloid PET findings. |
|  | Amyloid PET Negative | 30 |  |  |
| Jia (2021) | Dataset 1-Controls | 21 | CSF P-tau/Aβ42 | miRNAs are emerging as promising biomarkers in Alzheimer's disease (AD) research, offering high sensitivity for ultra-early detection during the asymptomatic stages. Studies suggest that predictive models incorporating specific panels of miRNAs (such as certain exosomal miRNA panels) can act as effective biomarkers to detect preclinical AD years before the onset of cognitive impairment. |
|  | Dataset 1-AD | 23 |  |  |
|  | Dataset 2-Controls | 216 |  |  |
|  | Dataset 2-AD | 190 |  |  |
|  | Dataset 3-Controls | 153 |  |  |
|  | Dataset 3-AD | 151 |  |  |
|  | Dataset 4-Controls | 139 |  |  |
|  | Dataset 4-AD | 155 |  |  |
|  | Dataset 4-aMCI | 55 |  |  |
| Jia (2022) | Control | 102 | P-tau/Ab42, CSF Ab42 , 500 pg/mL | A panel of six miRNAs serves as a potential blood-based biomarker for AD. More importantly, the predictive model incorporating these six miRNAs can detect preclinical AD five to seven years before the onset of cognitive impairment, achieving AUC ranging from 0.85 to 0.88 |
|  | Preclinical AD | 101 |  |  |
| Zhang (2017) | PiB positive | 16 | PiB-PET | Urine can serve as a source for fluid biomarkers, and NTP along with the MMSE are relevant indicators and clinical scales involved in AD research. |
|  | PiB negative | 14 |  |  |
| Shen (2023) | Aβ-CN | 68 | CSF Aβ40 CSF Aβ42 CSF t-tau CSF p-tau181 18F-FDG PET | GFAP is a promising biomarker across the AD continuum. Studies indicate that plasma GFAP levels can effectively distinguish preclinical AD from cognitively unimpaired controls. Furthermore, higher levels of plasma GFAP display predictive value for the risk of AD clinical progression and dementia, and it is correlated with other AD-related biomarkers. |
|  | Aβ+CN（preclinical Ad） | 34 |  |  |
|  | Aβ-MCI | 49 |  |  |
|  | Aβ+MCI（prodromal AD） | 34 |  |  |
| Guo (2023) | CU A−T− | 43 | CSF Aβ42，p-tau181；Aβ PET | AD continuum within the Chinese population, plasma GFAP and p-tau181 serve as important blood biomarkers that are closely associated with AD pathologies, such as amyloid positivity. Studies indicate that elevations in plasma GFAP may occur early in the disease process (i.e., during the preclinical stages). |
|  | CU A+T− | 17 |  |  |
|  | CU A+T+ | 17 |  |  |
|  | MCI+ | 36 |  |  |
|  | AD  dementia | 93 |  |  |
| Wang (2022) | AD | 26 | CSF Aβ42,  t-tau,  p-tau | AβPP, CSF biomarkers, and the MMSE are relevant indicators involved in AD research. Studies investigating these peripheral and fluid biomarkers contribute to the discovery of potentially feasible and effective screening tools for AD. |
|  | Non-AD | 24 |  |  |
|  | NC | 18 |  |  |
| Cheung (2021) | HC | 25 | PET-CT | Voxel-mirrored homotopic connectivity (VMHC) based on resting-state functional MRI is a neuroimaging biomarker. By combining VMHC values from relevant brain regions (such as the inferior frontal orbital gyrus and gyrus rectus), this technique can be utilized for the evaluation and diagnosis of Alzheimer's disease (e.g., identifying patients at the MCI-AD stage), achieving a diagnostic accuracy of 83% (with an AUC of 0.905). |
|  | MCI | 29 |  |  |
|  | AD | 16 |  |  |
| Lin (2023) | CU | 12 | Amyloid PET， Tau PET | Tau-PET imaging and related neuroimaging metrics (such as the standardized uptake value ratio, SUVR) are important neuroimaging biomarkers in AD continuum research, which can be utilized to assist in clinical diagnosis and are associated with patients' cognitive performance or function |
|  | AD-MCI | 20 |  |  |
|  | AD-D | 14 |  |  |
| Duan (2017) | HC | 16 | PiB PET | Neuroimaging technologies such as 11C-PiB PET can be utilized to detect and reflect Aβ pathology associated with AD. Studies have demonstrated that integrating optimally combined Regions of Interest (ROIs) from PiB-PET using machine learning techniques can serve as an effective neuroimaging biomarker for the evaluation and diagnosis of AD. |
|  | AD | 18 |  |  |
| Liu (2023) | AD | 30 | CSF Aβ42 or AβPET | CSF and serum can serve as sources for extracting and investigating fluid biomarkers for AD. In the search for novel diagnostic AD biomarkers, proteomic analysis is utilized to detect relevant markers, and these biomarker candidates are closely associated with key AD pathological pathways such as inflammation. |
|  | NC | 30 |  |  |
| 武晓丹 (2022) | PET + | 37 | 11 C-PIB PET/CT | CSF biomarkers (such as CSF p-tau181/Aβ42) demonstrate a high level of agreement (equivalent diagnostic accuracy) with amyloid PET imaging-based diagnoses, holding significant clinical value in the auxiliary diagnosis of AD. |
|  | PET - | 19 |  |  |
| Lu (2018) | Combined AD set-AD | 229 | Aβ PiB or CSF Aβ42, csf Tau, CSF Tau/Aβ42 | Blood-based biomarkers are being extensively investigated as potential tools for the early diagnosis of AD and its early stages, such as MCI. |
|  | MCI | 70 |  |  |
|  | Combined AD set-age-matched controls | 220 |  |  |
|  | Aβ positive AD set-AD | 40 |  |  |
| Chiu (2020) | Healthy control-derivation cohort | 67 | image biomarkers | Plasma biomarkers (such as plasma Aβ42) are utilized to assist in the differential diagnosis of early AD (including mild cognitive impairment due to AD and AD dementia), with participant diagnoses typically established according to clinical guidelines such as the NIA-AA criteria. |
|  | MCI due to AD-derivation cohort | 34 |  |  |
|  | AD dementia-derivation cohort | 59 |  |  |
| Li (2020) | Aβ _P | 78 | amyloid - PET | Features extracted from sMRI can serve as potential neuroimaging biomarkers for identifying preclinical AD. Based on data from cohorts such as the Sino Longitudinal Study on Cognitive Decline (SILCODE), researchers utilized machine learning algorithms like the support vector machine (SVM) to integrate stable high-frequency features derived from sMRI modalities (such as the large zone high-gray-level emphasis feature of the right posterior cingulate gyrus and the variance feature of the left superior parietal gyrus) to establish a classification model that effectively distinguishes preclinical AD from healthy controls. |
|  | Aβ _N | 105 |  |  |
|  | Cog_D | 24 |  |  |
|  | Cog_M | 27 |  |  |
| Xiao (2023) | Non-dementia | 1650 | Aβ-PET plasma p-tau217 plasma p-tau181 | Plasma biomarkers, such as p-tau217, p-tau181, and NfL, are broadly investigated in AD diagnosis and evaluation research. Among them, plasma p-tau217 demonstrates a strong performance in distinguishing Aβ+ individuals from Aβ- individuals. |
|  | AD dementia | 145 |  |  |
| Li (2025) | CU | 150 | Aβ-PET p-tau217 Aβ42 Aβ40 | In a Chinese population, the reference interval for plasma p-tau217 was determined to be between 0.006 and 0.47 pg/mL. In terms of diagnostic classification performance, plasma p-tau217 demonstrated exceptional accuracy in discriminating early-stage patients (MCI due to AD, MCI-AD) from cognitively unimpaired controls, achieving an AUC of 0.943. |
|  | MCI | 30 |  |  |
|  | AD | 60 |  |  |
|  | SIVD | 70 |  |  |
|  | FTLD | 40 |  |  |
| Hsieh (2025) | CAA | 59 | 11C-pib PET 18F-T807 PET p-tau217 Aβ42 Aβ40 t-tau | Plasma p-tau 217, along with its comparisons to other biomarkers (such as Aβ40, Aβ42, and total tau), is widely investigated in diagnostic classification and differential diagnosis research for AD and related pathologies, such as cerebral amyloid angiopathy (CAA). |
|  | AD | 19 |  |  |
|  | DPA | 22 |  |  |
| Zhao (2024) | SHAPE-CN | 10 | 18F-AV-1451 tau PET GFAP NfL | Composite biomarkers incorporating machine learning (specifically a random forest model) demonstrate excellent diagnostic utility in AD (such as identifying MCI due to AD). Studies indicate that by integrating eight critical variables (left amygdala SUVR, right amygdala SUVR, and left entorhinal cortex SUVR from tau PET, along with age, education, plasma NfL, plasma GFAP, and the plasma GFAP/NfL ratio), this model successfully differentiates MCI patients from healthy controls, achieving an AUC of 0.926. |
|  | SHAPE-MCI | 10 |  |  |
| Jiang (2024) | Cohort_1-CN | 317 | 11C-PiB PET | A plasma biomarker panel of 21 proteins (involving key pathological pathways such as neurodegeneration, inflammation, innate immunity, vascular function, and metabolic activity) demonstrates high efficacy in identifying early-stage AD (MCI due to AD), achieving an AUC of 0.8945. This assay shows strong diagnostic potential that is superior to traditional ATN (Amyloid, Tau, and Neurodegeneration) markers. Furthermore, the study reveals notable heterogeneity in early AD progression between ethnic groups: at the MCI-AD stage, the Chinese population is primarily characterized by inflammation and innate immune abnormalities, whereas the Hispanic population is mainly characterized by vascular dysfunction and innate immune abnormalities. |
|  | Cohort_1-MCI | 190 |  |  |
|  | Cohort_1-AD | 493 |  |  |
|  | Cohort_2-CN | 9 |  |  |
|  | Cohort_2-MCI | 13 |  |  |
|  | Cohort_2-AD | 25 |  |  |
| Chang (2025) | CU | 22 | Aβ PET Tau-PET | Plasma GFAP and Tau-PET (involving metrics such as the standardized uptake value ratio [SUVR] in regions like the amygdala) are important fluid and neuroimaging biomarkers in AD continuum research, including early stages such as MCI. Integrating and combining these multimodal biomarkers can effectively assist in the diagnostic classification of AD-related stages. |
|  | MCI | 46 |  |  |
|  | AD | 37 |  |  |
| Wang (2025) | CADS-CU | 44 | Aβ PET tau PET | In Chinese clinical and community cohorts, the plasma p-tau217/Aβ42 ratio has demonstrated high diagnostic accuracy in identifying abnormal Aβ PET and tau PET statuses. This performance not only outperforms other single blood markers (such as plasma p-tau217 alone, Aβ42/Aβ40, p-tau181, and p-tau181/Aβ42) but is also clinically equivalent to CSF diagnostic tests (like CSF p-tau181/Aβ42 and Aβ42/Aβ40), making it highly likely to replace expensive or invasive reference PET and CSF markers in routine clinical practice in the future. |
|  | CADS-MCI | 146 |  |  |
|  | CADS-Dementia | 201 |  |  |
|  | GHABS-CADS-CU | 72 |  |  |
|  | GHABS-MCI | 23 |  |  |
|  | GHABS-Dementia | 26 |  |  |
| Jiao (2025) | Cohort_1-AD | 275 | PiB-PET CSF Aβ42、Aβ40 | A combination of six plasma digital spectral biomarkers integrated with machine learning models demonstrates strong discriminative ability (AUC > 0.8) in distinguishing Alzheimer's disease (AD) patients from healthy controls. |
|  | Cohort_1-HC | 189 |  |  |
|  | Cohort_2-AD | 18 |  |  |
|  | Cohort_2-HC | 344 |  |  |
|  | Cohort_3-MCI | 151 |  |  |
| Wang (2025) | PiB+ MCI | 41 | 11C-PibPET | Neuroimaging biomarkers and their extracted relevant features (such as those from the posterior cingulate gyrus) have potential applications in the diagnostic and evaluative research of early AD, such as MCI due to AD |
|  | PiB- MCI | 18 |  |  |
|  | CUC | 49 |  |  |
| Xie (2022 | MCI | 38 | CSF Aβ42/Aβ40; CSF Aβ42/Aβ40 combined with APOE ε4 | the current study demonstrated that CSF core AD biomarkers had good performance in identifying different diagnostic groups and predicting Aβ PET positive or negative, which revealed the utility and universal applicability of CSF core AD biomarkers in Chinese dementia population. The findings may provide assistance in the diagnostic evaluation process for AD and be helpful in clinical practice and drug trials in China. |
|  | AD | 38 |  |  |
|  | Non-ADD | 12 |  |  |
| Gao (2023) | CN | 104 | plasma p-Tau181; serum GFAP; APOE ε4 + plasma p-Tau181+ serum GFAP | The study represents the first investigation to show consistent variations in AD core biomarkers among different regions of China’s Han population, supporting their use in AD diagnosis within the Chinese context. Additionally, we identified serum GFAP as an early indicator of brain atrophy and cognitive impairment, with the potential to predict a future decline in these domains. Moreover, our findings underscore the accurate discriminatory ability of plasma pTau and serum GFAP, either individually or in combination, in distinguishing Ab status. These results emphasize the potential utility of these minimally invasive biomarkers as valuable tools for AD diagnosis and monitoring in clinical trials. |
|  | MCI | 110 |  |  |
|  | AD | 208 |  |  |
|  | Non-ADD | 55 |  |  |
| Gao (2022) | CN | 96 | plasma p-tau181; plasma p-tau181/T-tau; sMRI (Isthmus of cingulate gyrus, middle temporal thinkness, inferior temporal thickness, Amygdaia) + APOE + plasma p-tau + plasma Aβ42/Aβ40 | The results revealed a universal applicability of the “A/T/N” framework in a Chinese population and established an optimal diagnostic model consisting of costeffective and non-invasive approaches for diagnosing AD. |
|  | MCI | 94 |  |  |
|  | EOAD | 107 |  |  |
|  | LOAD | 66 |  |  |
|  | Non-ADD | 48 |  |  |
| Wang (2025) | CADS-CU | 44 | plasma p-tau217; plasma p-tau181; plasma p-tau217/Aβ42; plasma p-tau181/Aβ42; plasma Aβ42/40 | Plasma p-tau217/Aβ42 has high performance in detecting cerebral AD pathologies, thus offering a promising tool for clinical diagnosis and community screening of AD. Lumipulse G plasma p-tau217 and the p-tau217/Aβ42 ratio accurately identified abnormal Aβ and tau PET statuses in both clinical and community cohorts; The performance of plasma p-tau217 and p-tau217/Aβ42 ratio were equivalent to CSF tests; Plasma p-tau217/Aβ42 ratio outperformed p-tau217 alone in identifying Aβ PET positivity, and this superiority is more obvious in the community cohort, suggesting an advantage in the early diagnosis of AD; Two cut points of p-tau217/Aβ42 were established in the Chinese population for clinical laboratory and community screening uses. |
|  | CADS-MCI | 146 |  |  |
|  | CADS-Dementia | 201 |  |  |
|  | GHABS-CU | 72 |  |  |
|  | GHABS-MCI | 23 |  |  |
|  | GHABS-Dementia | 26 |  |  |
| Lai (2024) | HC | 35 | plasma p-tau181; plasma GFAP; plasma p-tau217 | Plasma biomarkers measured with a technology independently developed in China demonstrate good performance in diagnosing AD dementia. Plasma p-tau217, in particular, demonstrates the highest diagnostic value and can be used for AD dementia screening of large populations. |
|  | MCI | 50 |  |  |
|  | AD | 30 |  |  |
| Jiang (2024) | HC | 110 | Enlarged choroid plexus（ChP)+HIP; combination of four neuroimaging measures (ChP + HIP+ LVV+ ECT) | ChP volume may be a novel neuroimaging marker associated with neurodegenerative changes and clinical AD manifestations. It could better detect the early stages of the AD and predict prognosis, and significantly enhance the differential diagnostic ability of hippocampus on the AD continuum. |
|  | MCI | 269 |  |  |
|  | AD | 228 |  |  |
| Zhong (2025) | SCABI-1 cohort amyloid negative | 106 | plasma p-tau217; plasma p-tau217/Aβ1-42 | Plasma p-tau217 and p-tau217/Aβ1-42 are effective diagnostic tools. However, patient demographics, apolipoprotein E ε4 status, and cognitive condition must be considered to improve specificity in the clinical practice. |
|  | SCABI-1 cohort amyloid positive | 76 |  |  |
|  | SCABI-2 cohort amyloid negative | 47 |  |  |
|  | SCABI-2 cohort amyloid positive | 31 |  |  |
|  | RCP cohort amyloid negative | 51 |  |  |
|  | RCP cohort amyloid positive | 49 |  |  |
| Lin (2025) | Aβ negative | 70 | plasma p-tau217; plasma p-tau217+APOE+GFAP; plasma p-tau181; plasma GFAP | Plasma p-tau217 and p-tau181 effectively predictAβ+among culturally different Asian populations. P-tau217 performed better, especially in the early stages of AD. Plasma p-tau217–based models reduced intermediate-risk classifications, suggesting fewer amyloid PET scans needed to confirm the diagnosis. |
|  | Aβ positive | 30 |  |  |

Table S2. Cohorts generating ≥2 studies reporting accuracy metrics on fluid or imaging biomarkers for early stages of AD

| **Cohorts** | Number of publications |
| --- | --- |
| SILCODE (the Sino-Longitudinal Cognitive Impairment and Dementia Study) | 11 |
| CLAS (the China Longitudinal Ageing Study) | 6 |
| BABRI (the Beijing Aging Brain Rejuvenation Initiative) | 5 |
| SAS (Shanghai Aging Study) | 5 |
| CANDI (the China Aging and Neurodegenerative Initiative) | 4 |
| CIBL (Chinese Imaging, Biomarkers, and Lifestyle) | 3 |
| CU-SEEDS (The Chinese University of Hong Kong - Screening for Early Alzheimer’s Disease) | 3 |
| NBH-ADsnp (the Nanjing Brain Hospital Alzheimer’s Disease Spectrum Neuroimaging Project) | 3 |
| CABLE (the China Aging and Neurodegenerative Disorder Initiative) | 2 |
| China COAST (the China Cognition and Aging Study) | 2 |
| MASHB (Mild Cognitive Impairment and Alzheimer’s Disease Study in Hebei Province) | 2 |
| SYS-AD (the Shandong Yanggu Study of Aging and Dementia) | 2 |
| The Shanghai Memory Study | 2 |

Table S3. Biomarkers with AUC>0.8 for diagnosing biomarker-confirmed MCI-AD

| Reference | Biomarkers | Detection technology | Cutoff | AUC | Sensitivity | Specificity | PPV | | NPV | Accuracy | Reference standard biomarkers | Simple size | Detection technology  (standard) | Reference standard biomarkers cutoff | Reference standard biomarkers company |
| --- | --- | --- | --- | --- | --- | --- | --- | --- | --- | --- | --- | --- | --- | --- | --- |
| Shen (2023) | Plasma GFAP | SIMOA | 86.21  pg/mL | 0.851 (0.764 - 0.937) | 88.2% | 83.6% | 73.2% | 93.3% | | NA | CSF Aβ42 or Amyloid PET | 700 | ELISA/18F-AV45 | <194.5 pg/mL /Visual positive | INNOTEST/ Siemens |
|  | Plasma GFAP + MMSE | SIMOA | - | 0.849 | - | - | - | - | | - |  |  |  |  |  |
|  | Plasma GFAP + Aβ42/40 + NfL | SIMOA | - | 0.848 | - | - | - | - | | - |  |  |  |  |  |
|  | Plasma GFAP + clinical indicators * | SIMOA | - | 0.857 | - | - | - | - | | - |  |  |  |  |  |
| Cheung (2021) | fMRI VMHC values Combined (5 brain regions) † | fMRI: VMHC values | 0.35 | 0.905 | 88.0% | 83.0% | 80.0% | 86.0% | | 83% | Amyloid PET | 83 | Aβ-PET (18-F Flutametamol) | Composite Z-score of SUVR > 0.62 | - |
| Li  (2022) ‡ | Plasma nEV Aβ42 | SIMOA | - | 85.67% | - | - | - | - | | - | Amyloid PET | 246 | 18F-AV45 (18F-florbetapir) | SUVR>1.18 | SIGNA, GE Healthcare |
|  | Plasma nEV Aβ42+*APOE* ε4 status | SIMOA | - | 87.39% | - | - | - | - | | - |  |  |  |  |  |
|  | Age+sex +*APOE* ε4 status+MMSE+ MoCA-B + AVLT N5 + AVLT N7 | SIMOA | - | 96.77% | - | - | - | - | | - |  |  |  |  |  |
| Li (2025) | Plasma p-tau217 | SIMOA | - | 0.943 | NA | NA | - | - | | NA | Aβ PET | 410 | - | Visual positive | - |
| Zhao (2024) | RandomForest-3 SUVRs + age + education + plasma NfL & GFAP & GFAP/ NfL | SIMOA 18F-AV-1451 PET | - | 0.926 | 0.939 | 0.856 | - | - | | 0.745 | Aβ PET | 20 | 18F-florbetapir PET | SUVR＞1.11 | - |
| Jiang (2024) | Plasma 21-protein biomarker | Proximity extension assay | - | 0.8945 | NA | NA | - | - | | NA | Aβ PET | 47 | 11C-PiB PET | SUVR＞1.4 | - |

*. Clinical indicators included age, sex, years of education, *APOE* ε4 allele.

†. Five combined brain regions include Inferior Frontal Operculum, Rolandic Operculum, Supplementary Motor Area, Inferior Frontal Orbital Gyrus, and Gyrus Rectus.

‡. Amyloid-PET is not necessary to diagnose aMCI or ADD, but in those subjects who had undergone PET examination, Aβ deposition had to be obvious.

Abbreviations: AUC, area under the curve; NPV, negative predictive value; PPV, positive predictive value; GFAP, Glial Fibrillary Acidic Protein; nEV, neuronal-derived extracellular vesicle; SIMOA, Single Molecular Array; VMHC, voxel-mirrored homotopic connectivity; AVLT N5, auditory verbal learning test-delayed memory; AVLT N7, auditory verbal learning test-recognition; PET, positron emission tomography; PiB-PET, Pittsburgh Compound-B PET; SUVR, standardized uptake value ratio; MRI, magnetic resonance imaging; fMRI, functional MRI; CSF, cerebrospinal fluid.

Table S4. Biomarkers for diagnosing preclinical AD with AUC>0.8

| **Short Title** | **Biomarkers** | **Detection technology** | **Cut-off** | **AUC** | **Sensitivity** | **Specificity** | **PPV** | **NPV** | **Accuracy** | **Standard biomarker** | **Sample size** | **Standard biomarker detection technology** | **Standard biomarker cutoff** | **Standard biomarker company** |
| --- | --- | --- | --- | --- | --- | --- | --- | --- | --- | --- | --- | --- | --- | --- |
| Sheng (2022) | Combined plasma Aβ markers, gut taxa, and cognitive tests | Meso Scale Discovery (MSD); Linear discriminant analysis effect size (LEfSe) method | 0.356 | 0.869 | 87.5 | 73.53 | NA | NA | NA | Amyloid PET | 88 | [18F]florbetapir (AV-45) PET | NA | NA |
|  | Gut microbiota-Panel 2 (taxa 1, taxa 2, and taxa 3) |  | 0.383 | 0.81 | 87.5 | 64.71 | NA | NA | NA |  |  |  |  |  |
| Cai (2023) | *APOE* + plasma Aβ42 + Plasma p-tau181 + Plasma NfL | SIMOA | NA | 0.81 | NA | NA | NA | NA | NA | CSF p-tau181/ Aβ42 & Aβ42 | 249 | ELISA | p-tau181/Aβ42>0.14 and Aβ42<500 pg/mL | INNOTEST |
| Jia (2021) | GAP43+neurogranin+SNAP25 + synaptotagmin 1+*APOE* | Co-immunoprecipitation (Thermo Fisher Scientific) | NA | 0.88 | NA | NA | NA | NA | NA | CSF p-tau/Aβ42 + T-tau/Aβ42 | 320 | ELISA | 0.14, 0.67 | INNOTEST |
| Jia (2022) | Blood exosomal miRNAs (six-miRNA panel) | NCode™ VILO™ miRNA qRT-PCR kit | NA | 0.852 | NA | NA | NA | NA | NA | p-tau/Ab42 + Ab42 | 203 | ELISA | >0.14, 500 pg/mL | INNOTEST |
|  | Blood exosomal miRNAs (six-miRNA panel) + *APOE* status |  | NA | 0.876 | NA | NA | NA | NA | NA | p-tau/Ab42 + Ab42 | 203 | ELISA | >0.14, 500 pg/mL | INNOTEST |
| Shen (2023) | Plasma GFAP | SIMOA | 68.59 ng/ml | 0.894 | 88.20% | 83.60% | 73.20% | 93.90% | NA | CSF Aβ42/PET | 700 | ELISA/18F-AV45 | <194.5 pg/mL /Visual positive | INNOTEST/ Siemens |
|  | Plasma GFAP + clinical indicators ^*^ |  | NA | 0.894 | NA | NA | NA | NA | NA |  |  |  |  |  |
|  | Plasma GFAP + Mini-Mental State Examination scores |  | NA | 0.898 | NA | NA | NA | NA | NA |  |  |  |  |  |
|  | Plasma GFAP+Aβ42/40+NfL |  | NA | 0.908 | NA | NA | NA | NA | NA |  |  |  |  |  |
| Li (2020) | Four sMRI features † | sMRI | NA | 0.863 | NA | NA | NA | NA | NA | Amyloid PET | 183 | Florbetapir F-18 (AV45) | Cutoff of >1.18/ Visual positive | SIGNA |

*. Clinical indicators included age, sex, years of education, *APOE* ε4 allele.

†. Four sMRI features were large zone high-gray-level emphasis (LZHGE) feature of the left posterior cingulate gyrus on sMRI combined with three stable high-frequency features on sMRI, including LZHGE feature of the right posterior cingulate gyrus, the variance feature of the left superior parietal gyrus and the coarseness feature of the left posterior cingulate gyrus.

Abbreviations: AUC, area under the curve; NPV, negative predictive value; PPV, positive predictive value; GFAP, Glial Fibrillary Acidic Protein; SIMOA, Single Molecular Array; ^1^H-MRS, Proton magnetic resonance spectroscopy; NAA, N-acetylaspartate; Cr, creatine; PET, positron emission tomography; PiB-PET, Pittsburgh Compound-B PET; MRI, magnetic resonance imaging; sMRI, structural MRI; CSF, cerebrospinal fluid.

Table S5. The recommended biomarkers for distinguishing amyloid-positive from amyloid-negative individuals

| **Short Title** | **Sample size** | **Biomarkers** | **Detection technology** | **AUC** | **Standard biomarker** | **Standard biomarker detection technology** | **Standard biomarker cutoff** | **Standard biomarker company** |
| --- | --- | --- | --- | --- | --- | --- | --- | --- |
| Xie (2022)  2/17 | 88 | CSF Aβ42/Aβ40 | SIMOA | 0.9235 | Amymoid PET | 18F-Florbetapir PET | Visual positive | 18F-Florbetapir (Siemens, Germany) |
|  |  | CSF Aβ42/Aβ40 combined with *APOE* ε4 |  | 0.9665 |  |  |  |  |
| Gao (2023)  3/3 | 477 | Plasma p-Tau181 | SIMOA | 0.839 | Amymoid PET | 18F-florbetapir PET (AV-45 PET) | NA | NA |
|  |  | Serum GFAP |  | 0.871 |  |  |  |  |
|  |  | *APOE* ε4 + plasma p-Tau181+ serum GFAP |  |  |  |  |  |  |
|  |  |  |  | 0.915 |  |  |  |  |
| Gao (2022)32/19  4/19 | 411 | Plasma p-tau181 | SIMOA | 0.856 | CSF Aβ | CSF Aβ42/Aβ40 | <0.0642 | NA |
|  |  |  |  | 0.849 | PET Aβ | Amymoid PET (18F-Florbetapir) | Visual positive | Biograph 16HR scanner (Siemens Healthcare) |
|  |  | Machine-learning Aβ-based logistic regression model (sMRI features + plasma biomarkers) ^*^ | GE DISCOVER 750w 3.0T MRI scanner; SIMOA | 0.914 | PET Aβ |  |  |  |
| Guo (2023)  2/7 | 206 | Plasma GFAP | SIMOA | 0.911 | CSF Aβ42 | ELISA | 194.50 pg/mL | INNOTEST |
|  |  | Plasma GFAP |  | 0.971 | Amymoid PET | amyloid (18FAV45) | Visual positive | Discovery 750, GE Healthcare |
|  |  | Plasma p-tau181 |  | 0.916 | Amymoid PET |  |  |  |
| Wang (2025) | 512 | Plasma p-tau217 | Lumipulse G1200 platform | 0.960 | Aβ PET | 11CPiB or 18FAV45 | Visual positive | NA |
|  |  | Plasma p-tau217/Aβ42 |  | 0.966 | Aβ PET |  |  |  |
| Lai (2024) | 115 | Plasma p-tau217 | Ultrasensitive Digital Immuno-Chip Technology | 0.99 | Aβ PET | 18F-AV45 | NA | NA |
| Jiang (2024) | 138 | Enlarged ChP | 3.0-T magnetic resonance scanner | 0.8063 | CSF Aβ42 | ELisa | Aβ42<650 pg/mL or Aβ42/40 ratio≤0.064 | Beijing Hightrust Diagnostics, Co, Ltd, China |
|  |  | ChP+HIP |  | 0.8155 | CSF Aβ42 |  |  |  |
|  |  | Combination of four neuroimaging measures (ChP + HIP+ LVV+ ECT) |  | 0.8257 | CSF Aβ42 |  |  |  |
| Zhong (2025) | 360 | Plasma p-tau217 | Lumipulse G1200 | 0.94-0.96 † | Amyloid PET or CSF Aβ1-42/Aβ1-40 | 18F-florbetapir PET Lumipulse | Visual positive Aβ1-42/Aβ1-40 0.059 | Fujirebio |
|  |  | Plasma p-tau217/Aβ42 |  | 0.96-0.97 † |  |  |  |  |
| Lin (2025) | 100 | p-tau217 | SIMOA | 0.966 | Amyloid PET | 18F-florbetapir PET | 37 Centiloid units | NA |
|  |  | p-tau217+*APOE*+GFAP |  | 0.968 |  |  |  |  |

*. The model included plasma p-tau, Aβ42/Aβ40, cortical thickness (inferior temporal and middle temporal), and brain volumetric measures (hippocampus and amygdala).

†. AUC ranges from three different cohorts. Plasma p-tau217: Southern China Aging Brain Initiative (SCABI)-1, AUC = 0.95 (95%CI: 0.91–0.98); SCABI-2, AUC = 0.96 (95%CI: 0.91–1); real‐world clinical practice (RCP), AUC = 0.94 (95%CI: 0.89–0.99). Plasma p-tau217/Aβ1-42: SCABI-1, AUC = 0.96 (95%CI: 0.93–0.99); SCABI-2, AUC = 0.97 (95%CI: 0.93–1); RCP, AUC = 0.96 (95%CI: 0.92–0.99).

Abbreviations: AUC, area under the curve; GFAP, Glial Fibrillary Acidic Protein; SIMOA, Single Molecular Array; ^1^H-MRS, Proton magnetic resonance spectroscopy; NAA, N-acetylaspartate; Cr, creatine; PET, positron emission tomography; PiB-PET, Pittsburgh Compound-B PET; MRI, magnetic resonance imaging; sMRI, structural MRI; CSF, cerebrospinal fluid; ChP, HIP, hippocampal volume; Choroid plexus; LVV, lateral ventricular volume; ECT, Episodic Memory Task.
